# P-1419. Vaccine failure of the live-attenuated vaccine after liver transplantation depends on the vaccine strain

**DOI:** 10.1093/ofid/ofae631.1594

**Published:** 2025-01-29

**Authors:** Munehiro Furuichi, Takuma Ohnishi, Mizuki Yaginuma, Yohei Yamada, Ken Hoshino, Tetsuo Nakayama, Masayoshi Shinjoh

**Affiliations:** Keio University School of Medicine, Shinjukuku, Tokyo, Japan; Keio University, Japan, Shinjuku-ku, Tokyo, Japan; Keio University, Japan, Shinjuku-ku, Tokyo, Japan; Keio University, Japan, Shinjuku-ku, Tokyo, Japan; Keio University, Japan, Shinjuku-ku, Tokyo, Japan; Kitasato University, Minato-ku, Tokyo, Japan; Keio University, Japan, Shinjuku-ku, Tokyo, Japan

## Abstract

**Background:**

A recent conditional recommendation suggests considering live-attenuated vaccines for solid organ transplant recipients, yet the conditions of their safe and effective administration remain unclear.

**Figure:**
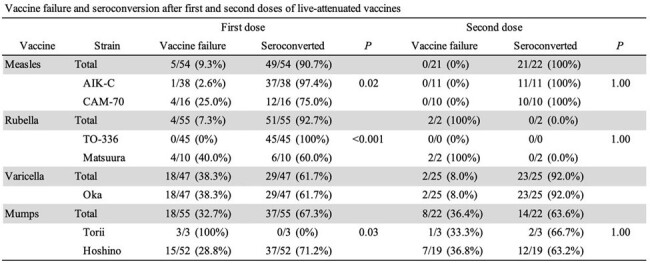

Primary vaccine failures after the first dose of live-attenuated vaccines (measles, rubella, and mumps) were significantly different between the vaccine strains.

**Methods:**

This prospective study, conducted at Keio University Hospital from 2002 to August 2023, gave live-attenuated vaccines to liver transplant (LT) recipients fulfilling the criteria for live-attenuated vaccines, including humoral and cell-mediated immunity. Patient background information, immunization date, vaccine strain, immunosuppressive agents at the time of vaccination, and antibody titers were collected. Factors related to primary and secondary vaccine failure were evaluated to enhance the effectiveness of the live-attenuated vaccine program after LT.

**Figure:**
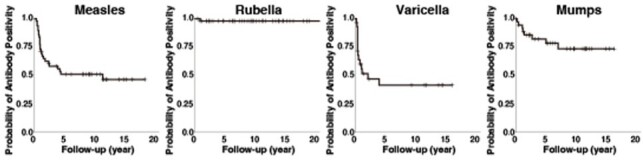

Kaplan–Meier curves for the proportion of remaining positive antibody titer after the first dose of indicated live-attenuated vaccine for post-liver transplant recipients.

**Results:**

Among 67 LT recipients, 54, 55, 47, or 55 received at least one dose of live-attenuated vaccine for measles, rubella, varicella, or mumps, respectively. Measles vaccine with the AIK-C strain exhibited significantly lower primary failure rates than the CAM-70 strain (1/38 vs. 4/16, odds ratio: 0.08, 95% confidence interval [CI]: 0.01–0.80, *p* = 0.02) (Table 1). No primary failures were observed with the TO-336 strain of rubella, whereas 4 of 10 LT recipients with the Matsuura strain of rubella did not seroconvert. For mumps, the Hoshino strain showed lower primary failure rates than the Torii strain (15/52 vs. 3/3, *p* = 0.03). Among seroconverted LT recipients after the first dose of live-attenuated vaccine, the proportion of remaining positive antibody titer is shown by Kaplan–Meier curves (Figure 1). In the years following the first doses of measles, varicella, and mumps vaccines, the proportion of remaining positive antibody titers decreased gradually. Furthermore, the AIK-C strain exhibited a significantly lower risk of secondary vaccine failure compared to the CAM-70 strain (hazard ratio: 0.29, 95% CI: 0.12–0.72, *p* = 0.01) (Figure 2).

**Figure:**
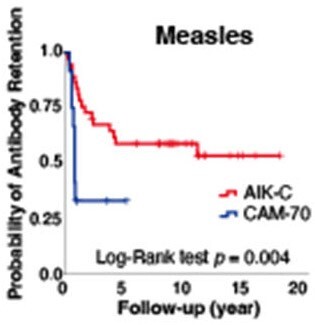

Kaplan–Meier curves for the proportion of remaining positive antibody titer after the first dose of measles vaccine for post-liver transplant recipients with Log-rank test for the vaccine strains.

**Conclusion:**

Vaccine strains are the most critical factor influencing primary and secondary vaccine failure in post-transplant live-attenuated vaccination.

**Disclosures:**

**Masayoshi Shinjoh, MD, PhD**, Meiji Seika Pharma Co., Ltd: Honoraria|Mitsubishi Tanabe Pharma Corporation: Honoraria|MSD K.K.: Honoraria|Pfizer Japan Inc: Advisor/Consultant|Shionogi & Co., Ltd: Honoraria

